# Molecular identification of *Anopheles squamosus* (Diptera: Culicidae) using internal transcribed spacer 2

**DOI:** 10.21203/rs.3.rs-7278578/v1

**Published:** 2025-08-19

**Authors:** Valerie T. Nguyen, Renee L. M. N. Ali, Bianca C. Burini, Dalia S. Dryden, Megan A. Riddin, Kochelani Saili, Edgar Simulundu, Lawrence E. Reeves, Douglas E. Norris, Yoosook Lee

**Affiliations:** University of Florida; The Johns Hopkins Malaria Research Institute, Johns Hopkins Bloomberg School of Public Health, Johns Hopkins University; University of Florida; University of Florida; University of Pretoria; Macha Research Trust; Macha Research Trust; University of Florida; The Johns Hopkins Malaria Research Institute, Johns Hopkins Bloomberg School of Public Health, Johns Hopkins University; University of Florida

**Keywords:** Anopheles squamosus, molecular diagnosis, internal transcribed spacer, mosquito, malaria

## Abstract

**Background:**

*Anopheles squamosus* is a widespread mosquito species in sub-Saharan Africa. It is a potential vector for human malaria parasites and has been found naturally infected with *Plasmodium falciparum* and *Plasmodium vivax*. Morphological identification is challenging even with pristine specimens and current molecular methods such as the use of the internal transcribed spacer 2 (ITS2) polymerase chain reaction (PCR) cannot distinguish *An. squamosus* from morphologically similar *Anopheles species*.

**Methods:**

Multiple alignments of previously published ITS2 contig sequences in NCBI from *An. squamosus* and *An*. species 11 and 15, were used to identify candidate ITS2 regions for primer design. We evaluated six sets of primers overall for specificity of species identification. The one set with *An. squamosus* species-specific amplification was tested using 78 specimens from Zambia and South Africa.

**Results:**

A new assay consisting of a forward (ITS2-ASQ-R10, 5’-CCC TCG AAG GGT GCT GTG-3’) and reverse (ITS2-ASQ-R10 5’-AAT CCA CGG TGT GAT GGC-3’) primer reliably (> 94.8%) amplified an ITS2 fragment of 301bp length for *An. squamosus*. The *An. squamosus*-specific primer set can be multiplexed with existing ITS2 assays frequently used for anopheline species identification.

**Conclusions:**

The development of this robust PCR assay for *An. squamosus* is vital to accurate identification of this species in malaria vector surveillance efforts. Improved understanding of the anopheline community composition will lead to better targeted methods of vector eradication and malaria prevention. In addition, investigating host association and malaria transmission can be facilitated with this assay by correctly identifying *An. squamosus*. Applying genomic tools to correctly identified anopheline species may lead to the discovery of genetic factors that influence its behavior and new innovations in malaria elimination.

## Introduction

With global concerted efforts to reduce the burden of malaria, the World Health Organization (WHO) created the Global Technical Strategy for Malaria 2016–2030 (GTS) with the goal to reduce malaria incidence and mortality rates by 90% in 35 countries during this period [1]. These efforts have led several regions within Africa to reach a pre-elimination stage of malaria transmission. Pre-elimination for a region is defined as a population with either a Rapid Diagnostic Test (RDT) positivity rate below 5% annually or a parasite positivity rate lower than 5% among those with fever [2]. Countries and regions that have met these pivotal milestones towards malaria elimination include Cape Verde, central Senegal, Guinea-Bissau, Isle of Príncipe, and southern Zambia [3–9].

In some cases, secondary malaria vectors have been implicated in transmission of malaria parasites in pre-elimination areas. These secondary vectors include *Anopheles vaneedeni* and *An. parensis* in areas of southern Africa and *An. coustani* and *An. ziemanni* in central Africa [28–31]. In Zambia, anopheline species like *An. squamosus*, *An. rufipes*, and *An. coustani* are of increasing concern as vectors for malaria in pre-elimination areas after reductions in populations of the primary vector species [11–15].

*Anopheles squamosus* is an anopheline species that is common and collected in abundance across sub-Saharan Africa [16] including Zambia. Previous work suggested that this species could be a species complex based on chromosome inversion polymorphism data [17]. In the adult stage, this species is morphologically identical to *An. cydippis* and difficult to distinguish molecularly from other unnamed Anopheles species like *Anopheles* sp. 11 and *Anopheles* sp. 15, cryptic species related to *An. squamosus* [18, 19]. There is no morphological data for *An. sp*. *11* and *15* to compare with *An. squamosus* to date. Therefore, morphological identification for *An. squamosus* can be unreliable and currently requires sequencing of fragments of the cytochrome c oxidase subunit I (COI) and/or internal transcribed spacer 2 (ITS2) genes for species confirmation [4].

While COI is often used for species identification [20–22], mitochondrial markers are often insufficient to delineate mosquito species within closely related species groups in anopheline mosquitoes [23–25]. For this reason, ITS2 regions on the X chromosome of *Anopheles* species are commonly used for molecular species identification [26, 26–28]. Internal transcribed spacer 2 is used primarily for parsing cryptic species in anopheline complexes, such as species of the *An. maculatus* complex, *An. maculipennis* complex, *An. quadrimaculatus* complex, *An. fluviatilis* complex, *An. crucians* complex and the *An. nili* group [25–29].

The challenge in applying this method to identification of *An. squamosus* is that the currently available primer sets used in ITS2 identification assays for anopheline species do not reliably produce PCR products from *An. squamosus* DNA templates [13]. Inability to accurately identify *An. squamosus* limits the capacity of routine surveillance to detect the presence of this potential malaria vector and may lead to misidentification with species not implicated in malaria transmission [24]. The lack of a reliable molecular identification tool for this species also hinders further research. Application of genomic tools to accurately identified species may lead to the discovery of genomic regions responsible for parasite infection, insecticide resistance, host choice, and other traits relevant to pathogen transmission [14–18]. In this study, a new reliable ITS2 PCR assay is introduced that distinguishes *An. squamosus* from other sympatric *Anopheles species*.

## Materials and Methods

### Sample Collection and DNA Preparation

*Anopheles squamosus* specimens from Zambia and South Africa were used for this study. Specimens from Macha in Choma District, Southern Province, Zambia (16.42181°S, 177.20417°W) were collected in January 2023 using a CDC light trap placed inside animal pens or human dwellings upon permission from homeowners. Specimen collection in Limpopo Province, South Africa (23.4013° S, 29.4179° E) was conducted using CO_2_-baited tent traps and sweep nets. Morphological identification was done using a morphological key to African anopheline species [17]. Samples were stored in 80% ethanol and refrigerated at 4°C until DNA extraction. To extract DNA from individual Zambian mosquitoes, a magnetic bead-based protocol as described by Chen et al. [19] was used. The DNA from South African samples was extracted with the Macherey-Nagel NuceloSpin Kit following the manufacturer’s instructions (Düren, Germany).

### Primer Design

Previously published ITS2 contig sequences (National Center for Biotechnology Information GenBank Accession number: MK592048, MK592075, OQ241725, MK592071) were obtained for *An*. sp. 11, *An*. sp. 15, and *An. squamosus* for primer design [30]. These sequences were selected because of their high sequence similarity (between 73.33% and 90.82%) to the available *An. squamosus* ITS2 sequences in GenBank, which allowed primer design that was specific for the target species. Of note, there is currently no sequence data available for *An. cydippus*. Multiple sequence alignment of these ITS2 sequences was conducted in Geneious Prime (version 2023.1.2) [20], which illuminated candidate ITS2 regions where *An. squamosus*-specific amplificon could be achieved. The consensus sequences of the multiple alignment were used as input sequences for primer design using Primer-BLAST [31]. A target amplimer range was set between 290 to 315 bp, so it can be multiplexed with the existing ITS2 sequence, which produces bands > 400bp for other *Anopheles* species. Programs such as MPprimer (version 3.1) [21] were used to test the primer compatibility for multiplex PCR. Six candidate primer sets (using three forward primers and reverse primers) were identified as compatible primers and used for assay validation (Table 1).

### PCR Validation

A 25 μL PCR mixture was prepared for each mosquito specimen to contain 2–5 μL of extracted DNA template, 2.5 μL of 10× PCR buffer, 2.0 μL of 10 mM dNTPs mix, 0.4 μL of Promega GoTaq Taq DNA polymerase (5 U/μl; Madison, WI, USA), 0.3 μL of each forward and reverse primer (10 μM), and 14.5–17.5 μL of PCR-grade water. Assay validation was performed on *An. squamosus*, *An. sp. 11, An. sp. 15, An. stephensi, An. arabiensis, An. gambiae* s.s., and *An. funestus* s.s to screen for primer specificity. The robustness of the PCR amplification for *An. squamosus* was evaluated on 78 replicates of individual specimens of *An. squamosus*.

PCR conditions were as follows: initial denaturation at 94°C for 2 min followed by 39 cycles of 94°C for 30 s, 57.6°C for 30 s, and 72°C for 40 s. Then a final extension step of 72°C for 10 min before being held at 4°C. Amplification of a PCR product of the expected size range was confirmed by electrophoresis on a 1.5% agarose gel.

**Figure 1:**
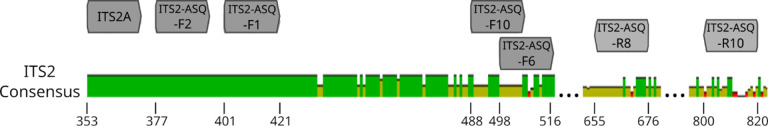
Primer position relative to the consensus ITS2 sequences of *An. squamosus*

A total of four new forward primers (ITS2-ASQ-F1, -F2, -F6, and -F10) and two new reverse primers (ITS2-ASQ-R8 and -R10) were designed (Fig. 1, Table 1). Six combinations of these primers were tested for species-specific amplification.

### Cocktail PCR Validation

The new primer sets were assessed in a multiplexed PCR cocktail with existing *Anopheles* ITS2 primers (UV [32] or ITS2A [33], ITS2b [33], ITS-ASQ-F10, ITS-ASQ-R10) on individual specimens of *An. squamosus, An. sp. 11, An. sp. 15, An. stephensi, An. arabiensis, An. gambiae* s.s., and *An. funestus* s.s. These *Anopheles* species were selected due to their overlapping distribution across Africa. The PCR conditions were the same as described above.

## Results

**Figure 2:**
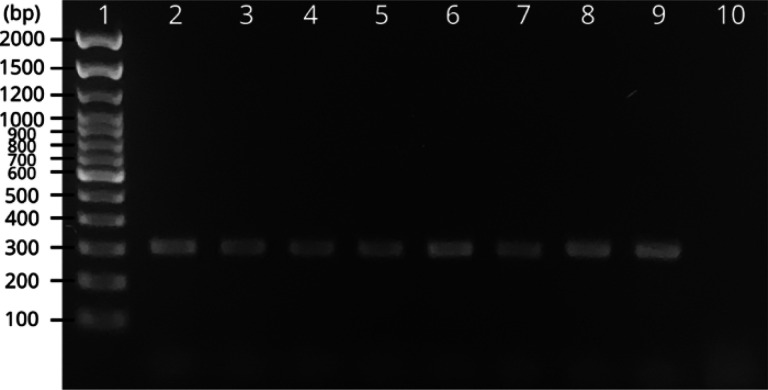
ITS2 PCR results for *An. squamosus* amplification ~300 bp. Lane 1: ladder. Lanes 2–9: eight different individual *An. squamosus* specimens (one specimen per lane). Lane 10: negative control.

Among the primers tested, the combination of ITS2-ASQ-F10 and ITS2-ASQ-R10 produced *An. squamosus*-specific amplificons (Fig. 2) for 74 of 78 individual *An. squamosus* specimens (72 from Zambia, six from South Africa) evaluated. With only four reactions failed to produce an amplicon (5.1% false negative rate), this assay demonstrated a robust (> 94.9%) success rate.

**Figure 3:**
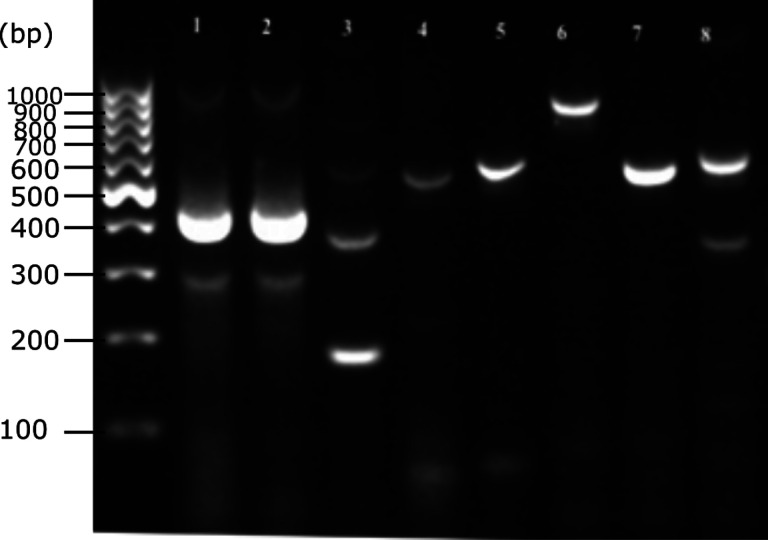
Multiplexed ITS2 PCR results showingITS-ASQ-F10 and ITS-ASQ-R10 primers used together with ITS2A and ITS2B primers. Lanes 1–2: *An. squamosus*. Lane 3: *An*. sp. 11. Lane 4: *An*. sp. 15. Lane 5: *An*. *stephensi*. Lane 6: *An. arabiensis*. Lane 7: *An. gambiae* s.s. Lane 8: *An. funestus* s.s

The new ITS2-ASQ-F10/ITS2-ASQ-R10 primer set was successfully used with existing *Anopheles* ITS2 PCR primers (ITS2A and ITS2B) that have been typically used for *Anopheles* species identification [33] (Fig. 3). Testing of the multiplexed ITS2 PCR showed unique amplimer sizes for each *Anopheles* species with *An. squamosus* distinct from the anopheline mosquitoes included here.

## Discussion

In the absence of morphological discrimination, an *An. squamosus*-specific molecular tool is needed to positively identify this species. Compatibility and the ability to multiplex with existing PCR-based assays [34] is ideal to reduce both costs and effort, making the approach viable for implementation as a routine malaria vector surveillance method in Africa.

This study resulted in a primer set that can robustly (> 94.9%) amplify a fragment of the *An. squamosus* ITS2 gene when multiplexed with the standard ITS2A/ITS2B primers. The size of the amplicon was 301 bp, which was specific to *An. squamosus* against all the other African *Anopheles* species evaluated within this study. Other *Anopheles* species routinely caught in collections with *An. squamosus* produce different sized ITS2 amplicons from the ITS2A and ITS2B primers (Fig. 3). For example, members of the *An. gambiae* complex produce an amplicon near 600 bp, while members of the *An. funestus* group produce an amplicon of 850 bp. *Anopheles rufipes, An. maculipalpis*, and *An. pretoriensis* generate indistinguishable amplicons of approximately 500 bp when using the ITS2A and ITS2B primers. The reaction could be further optimized using a touchdown PCR to reduce the unspecific binding and increase specificity.

With an understanding of anopheline community composition, species-specific targeted and effective methods of control may be better implemented. For example, indoor residual spraying and long-lasting insecticidal netting are widely used in malaria-endemic areas, as they have been most effective at targeting the behavior of endophilic and endophagic primary vectors (WHO 2023, Bhatt 2015). These efforts have resulted in reductions of principal malaria vectors such as *Anopheles gambiae s.l*. in Kenya, Tanzania, and Zambia [15, 35, 36]. However, these intervention methods do not consider foraging and resting behaviors of secondary vectors that may be transmitting malaria at low levels [10, 11].

This new *An. squamosus*-specific assay was evaluated on specimens from a relatively narrow geographic range of the known *An. squamosus* distribution; Zambia and South Africa. Therefore, this assay should be further evaluated on *An. squamosus* from a much broader geographic region to assess accuracy and robustness, especially for geographically isolated populations such as Madagascar, where *An. squamosus* is abundant [37]. Moreover, Coetzee [17] suggested that *An. squamosus* is likely to be a species complex based on chromosome inversion polymorphisms. If this is true, the ITS2 PCR alone may not be sufficient to delineate species within this complex and may need further refinement, as has been demonstrated for the *An. gambiae* complex [38]. In addition, *An. cydippis* and additional species that are sympatric to *An. squamosus* should be evaluated to ensure that this new ITS2 primer set is indeed species-specific.

This assay allows the more robust surveillance of *An. squamosus* in Africa. Though cryptic with other taxa as adults, this species can be morphologically differentiated as 4th instar larvae. Unfortunately, earlier instars may not have developed distinguishable features. In addition, common methods of trapping adult anopheline such as CDC miniature light traps may damage specimens and lose key features that are used to distinguish them morphologically, such as wings, legs, and scales [19]. DNA-based tools, such as the ITS2 assay, avoid these challenges, providing robust identification regardless of life stage or specimen quality with minimal tissue input [39].

This assay opens new opportunities for investigating the role of *An. squamosus* in malaria transmission. By exploiting the genetics of vectors and building resources to study them, we can identify genes that can be tied to vector competence. For example, in *Anopheles arabiensis*, there was an identified genetic component that influenced its host choice and behavior [40]. Alleles linked to the 2Rb and/or 3Ra inversions were linked to cattle-feeding preferences in *An. arabiensis*. The link between genetics and host preference is vital to further assess how *An. squamosus* is contributing to disease transmission with a new and accurate method of identification of this species.

The development of this ITS2 primer for *An. squamosus*, using the methods detailed here, can be applied to other understudied species and secondary vectors of malaria that do not have reliable methods of identification. For example, *An. pharoensis* is also found to be infected with *P. falciparum*, but does not reliably amplify in currently available ITS2. This assay shows repeatability, reproducibility, specificity, and a high limit of detection (> 95% amplification of biological replicates used), showing these primer sets provide a robust method for the detection of *An. squamosus*. This framework will allow for accurate and robust monitoring of secondary vectors of malaria.

## Figures and Tables

**Table 1:** A list of candidate primer sets used to assess their specificity to *An. squamosus*. The bold font indicates the primer set used for further testing based on the reliability of *An. squamosus*-specific amplification. In the primer name, ITS2A refers to a universal primer for *Anopheles* [33] (sometimes denoted as UV [32]). F and R refer to the forward primer and the reverse primer.

Primer pairs	Forward primer	Reverse primer	Amplimer length (bp)
ITS2A + ITS2-ASQ-R8	5’-TGTGAACTGCAGGACACAT-3’	5’-TCAACGTACCACACTTGACG-3’	301
ITS2-ASQ-F1 + ITS2-ASQ-R8	5’-CATCGGACGTTCTAACACGA-3’	5’-TCAACGTACCACACTTGACG-3’	253
ITS2-ASQ-F2 + ITS2-ASQ-R8	5’-TCGACACGTTGAACGCATA-3’	5’-TCAACGTACCACACTTGACG-3’	277
ITS2A + ITS2-ASQ-R10	5’-TGTGAACTGCAGGACACAT-3’	5’-AATCCACGGTGTGATGGC-3’	436
ITS2-ASQ-F6 + ITS2-ASQ-R10	5’-GTGCTGTGGGACAATCCAC-3’	5’-AATCCACGGTGTGATGGC-3’	291
**ITS2-ASQ-F10 + ITS2-ASQ-R10**	5’-**CCCTCGAAGGGTGCTGTG**-3’	5’-**AATCCACGGTGTGATGGC**-3’	**301**

## Data Availability

All data generated or analyzed during this study are included in this article.
